# Supplementary figures and images for: Effects of Baru Almond Oil (Dipteryx alata Vog.) Treatment on Thrombotic Processes, Platelet Aggregation, and Vascular Function in Aorta Arteries
Source: Nutrients. 2022 May 18;14(10):2098. doi: 10.3390/nu14102098 (PMC9143840; doi:10.3390/nu14102098)

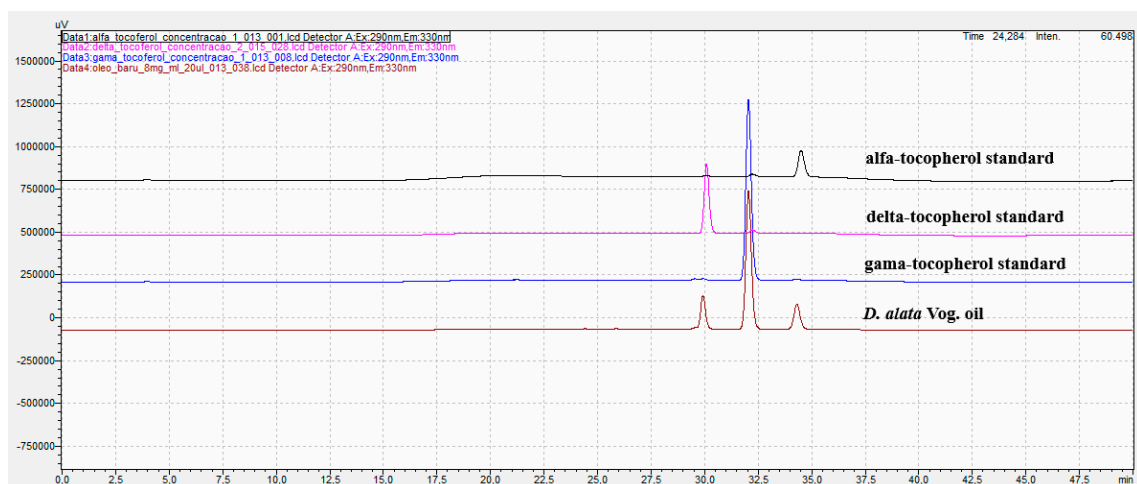

**Supplemental Figure S1.** Typical chromatogram of the separation of tocopherols in Baru oil.

Supplement: Supplementary file 1 [file nutrients-14-02098-s001.zip › nutrients-1695474-supplementary.pdf]
